# A Low-Cost Microfluidic-Based Detection Device for Rapid Identification and Quantification of Biomarkers-Based on a Smartphone

**DOI:** 10.3390/bios13070753

**Published:** 2023-07-22

**Authors:** Chonghui Yang, Yujing Yang, Gaozhen Zhao, Huan Wang, Yang Dai, Xiaowen Huang

**Affiliations:** State Key Laboratory of Biobased Material and Green Papermaking, School of Bioengineering, Qilu University of Technology, Shandong Academy of Sciences, Jinan 250353, China; yangchonghui2022@126.com (C.Y.); 10431211246@stu.qlu.edu.cn (Y.Y.); 10431210986@stu.qlu.edu.cn (G.Z.); 10431221233@stu.qlu.edu.cn (H.W.); 10431221222@stu.qlu.edu.cn (Y.D.)

**Keywords:** smartphone, fluorescence, glass capillary, biomarkers, RGB

## Abstract

The sensitive and rapid detection of microsamples is crucial for early diagnosis of diseases. The short response times and low sample volume requirements of microfluidic chips have shown great potential in early diagnosis, but there are still shortcomings such as complex preparation processes and high costs. We developed a low-cost smartphone-based fluorescence detection device (Smartphone-BFDD) without precision equipment for rapid identification and quantification of biomarkers on glass capillary. The device combines microfluidic technology with RGB image analysis, effectively reducing the sample volume to 20 μL and detection time to only 30 min. For the sensitivity of the device, we constructed a standard sandwich immunoassay (antibody–antigen–antibody) in a glass capillary using the N-protein of SARS-CoV-2 as a biological model, realizing a low limit of detection (LOD, 40 ng mL^−1^). This device provides potential applications for different biomarkers and offers wide use for rapid biochemical analysis in biomedical research.

## 1. Introduction

Early diagnosis is recognized as an effective strategy for the prevention and control of disease progression [1]. Although traditional diagnostic methods such as liquid biopsy [2,3,4], imaging examinations [5], and nucleic acid testing [6] have played a positive role, they are still facing problems such as long time consumption, high cost, and poor specificity. With the rapid development of detection technology, protein-based biomarkers [7,8,9], which are easier to isolate and can reflect more information about the actual physiological state of cells or tissues, are becoming faster, more convenient, and more cost-effective models for disease detection. The detection methods for protein biomarkers in the blood include enzyme-linked immunosorbent assay [10], chemiluminescence immunoassay [11], radiometric analysis [12], immunofluorescence analysis [13,14], etc. Among them, compared with other methods, immunofluorescence analysis has the advantages of convenient operation, stable signal, and no radioactive pollution. It is very suitable for the rapid quantitative detection of biomarkers. Immunofluorescence analysis is generally performed using fluorescence immunoassay analyzers [15] and fluorescence microscopes [16,17], which have the advantages of sensitivity and accuracy. However, there are problems such as large size, complex structure, and high price, which make it unsuitable for diagnosis in many environments. Therefore, there is an urgent necessity to develop a low-cost and portable fluorescence detection device.

With the rapid advances in imaging and sensing technology, smartphones have been equipped with the ability to capture images clearly and quickly, and they have been employed as detectors in fields such as pathogen imaging [18], absorbance detection [19], and chemiluminescence [20]. Smartphone-based detection devices have been widely utilized for biomedical applications [21,22], including disease diagnosis, bacteria detection, virus detection, and food technology. The miniaturization and portability of smartphone-based detection devices have allowed for the avoidance of large-scale equipment such as fluorescence spectrometers and microplate readers. Meanwhile, smartphones can store and share data in real time, providing new opportunities for developing low-cost, portable fluorescence detection devices [23,24,25]. However, early disease diagnosis requires the detection of low concentrations of biomarkers, which provides a challenge for smartphone-based fluorescence devices [26]. Therefore, there is an urgent requirement for a detection technique that can enhance its sensitivity. Fluorescence is light in a specific wavelength range, and it exhibits a specific color, showing different gray values in the three RGB channels [27]. Therefore, analyzing the relationship between biomarkers and the gray values of the RGB channels of the fluorescence image provides a new way for fluorescence quantification. Some scholars utilized cost-effective functional components to fabricate multiple portable detection devices. The sensitivity of fluorescence detection has been enhanced by using methods such as image stacking image analysis and improved fluorescence detection through capillary array, specifically targeting smartphones and webcams. Portable detection devices involve multiple functional components of different specifications, but the limited flexibility in assembling these multifunctional components becomes a challenge in achieving integrated devices. Burgeoning 3D-printing technology possesses the characteristics of low cost and high flexibility, providing a new strategy for flexibly printing support structures for functional components and manufacturing portable detection devices.

Microfluidic technology enables precise control and manipulation of samples through miniaturized fluidic channels and microscale operations [28]. It has the significant advantages of high accuracy, short time, and low cost, and it has great potential for early disease diagnosis [29,30,31]. Furthermore, the integration of smartphones with advanced computing capabilities and microfluidic technology allows for the integration and miniaturization of laboratory functions, enabling real-time on-site analysis of samples. Currently, smartphone-based detection devices incorporating microfluidic technology have been widely utilized for the detection of various biomarkers [32,33,34,35]. Traditional microfluidic chips are mainly made of materials such as PDMS and PMMA, which have good biocompatibility and high light transmission [36]. Although these materials are suitable for immunofluorescence analysis, the preparation process is complex and requires the use of sophisticated equipment. Therefore, it is necessary to find new microfluidic carriers with high light transmission and low cost.

Hence, we developed a low-cost smartphone-based fluorescence detection device (Smartphone-BFDD) without precision equipment for rapid identification and quantification of biomarkers on glass capillaries. In the developed system, 3D-printing technology and modular assembly strategy were used to miniaturize the detection system, mainly consisting of four main modules: fluorescence detection, excitation, camera, and power. The optical system of Smartphone-BFDD uses an orthogonal structure and optimizes the angle between the light source and capillary to reduce the impact of excitation light on fluorescence. Fluorescence images are captured using a smartphone (iPhone 11, Apple, Cupertino, CA, USA) as a model, and image J (version 1.53a) is used to analyze the gray values of the three channels to determine their intensity. The cheap glass capillary was employed as a carrier for microsamples, eliminating the need for preparing microfluidic chips and enabling automatic sample injection by the capillary phenomenon. To evaluate the optimal parameters of the smartphone for capturing fluorescent images, we tested the effect of shutter speed and photosensitivity (ISO) on the signal-to-noise ratio (SNR) by employing the BSA-FITC as a model. Meanwhile, the N-protein of SARS-CoV-2 was used as a detection model to evaluate the actual detection performance of Smartphone-BFDD for biomarkers under the optimal parameters.

## 2. Materials and Methods

### 2.1. Materials

(3-Aminopropyl)triethoxysilane (APTES, 99%) was purchased from Shanghai Aladdin Biochemical Technology Co., Ltd (Shanghai, China). N-Hydroxysuccinimide (NHS), H_2_O_2_ (30%), KOH, and glutaraldehyde (GA, 25%) was purchased from Sinopharm Chemical Reagent Co., Ltd. H_2_SO_4_ was purchased from Yantai Far East Fine Chemical Co., Ltd (Yantai, China). Phosphate-buffered saline (PBS) solution was purchased from Cytiva. Bovine serum albumin labelled with fluorescein isothiocyanate (BSA-FITC) was purchased from Dalian Meilun Biotechnology Co., Ltd. Glass capillary (0.5 mm in diameter, 8 cm in length) was purchased from the Instrument Plant of West China Medical University (Chengdu, China). Mouse anti-SARS-CoV-2 NP monoclonal antibody-1 (Capture antibody), mouse anti-SARS-CoV-2 NP monoclonal antibody-2 (detection antibody), and recombinant human SARS-CoV-2 NP protein (N-protein) was purchased from Nanjing Baikang Biotechnology Co., Ltd (Nanjing, China). Green fluorescent microspheres was purchased from Huge Biotechnology Co., Ltd (Shanghai, China). MES, Free acid, HEPES was purchased from Coolaber. EDC•HCL was purchased from Solarbio Life Science Co., Ltd (Beijing, China). Green fluorescence microspheres (100 nm) were purchased from Huge Biotechnology. Bovine serum albumin was purchased from Sigma-Aldrich (St. Louis, MO, USA).

### 2.2. Design of the Smartphone-BFDD

**Figure 1:**
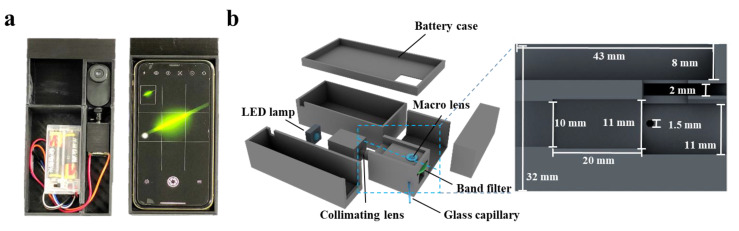
(**a**) Photograph of the smartphone-based fluorescence detection device (Smartphone-BFDD). (**b**) Schematic illustration of the Smartphone-BFDD.

Cinema 4D R20 (Maxon, Bad Homburg, Germany) was used to design the Smartphone-BFDD, which was printed by a 3D printer (i3 Mega S, Anycubic, Shenzhen, China). The photograph of Smartphone-BFDD is shown in Figure 1a; the overall structure mimics an orthogonal fluorescence microscope, and a smartphone was used to capture and display fluorescent images. In the 3D schematic illustration of PBTD in Figure 1b, PBTD consisted of a low-power LED lamp (1W, 470–475 nm), a collimating lens, a bandpass filter (500–700 nm, 1.7 mm), a macrolens, a battery case (3 V), a smartphone (iPhone 11, Apple, Cupertino, CA, USA), and 3D-printed cases. The macrolens was used to assist the smartphone in capturing fluorescent images and placed in a square groove (8 × 43 × 23.2 mm). The collimating lens is used to convert the flood light into parallel light, which can effectively alleviate the light interference and low light intensity in the area caused by the LED flood light. Meanwhile, the parallel light is not perpendicular to the glass capillary but crosses it at 120°, which reduces the generation of refracted light.

### 2.3. Fluorescence Quantification of BAS-FITC

**Figure 2:**
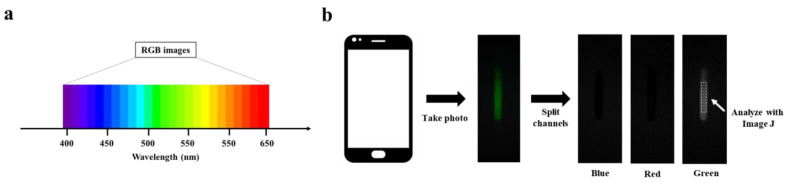
RGB image analysis: (**a**) Correspondence between RGB image and wavelength. (**b**) Schematic of RGB image analysis.

The 7.16 mg mL^−1^ BSA-FITC solution was diluted to a concentration range of 0~22.375 μg mL^−1^ with PBS buffer solution. An amount of 20 μL of BSA-FITC dilution was injected into a clean glass capillary, which was then placed in PBST for capturing fluorescence images of the BSA-FITC dilution. The processing steps are shown in Figure 2b. To capture high-quality fluorescent images, the smartphone needs to be adjusted for the right shutter speed and photosensitivity (ISO) before capturing the image. The fluorescent image is then divided into three channels: blue, green, and red. Finally, an area is selected in the green channel image for analyzing its fluorescence intensity.

### 2.4. Modification of Glass Capillary

The glass capillary was modified in three steps: (a) hydrophilic modification; (b) modification of APTES; and (c) modification of GA. The steps of modification are shown in Figure S1. First, the glass capillary was immersed in piranha solution (H_2_O_2_:H_2_SO_4_ 2:3) and 1 M KOH for 15 min, respectively. Then, the glass capillary was thoroughly cleaned with ultrapure water three times and dried with nitrogen. The clean glass capillary produces a large number of hydroxyl groups, and the APTES solution (ethanol: APTES 9:1) should be injected in time. The glass capillary with APTES solution was heated at 50 °C. After a 3 h reaction, the glass capillary was thoroughly cleaned by ethanol and crosslinked for 1 h at 125 °C. The 600 μL GA diluent (2.5%, PBS) is continuously injected into the glass capillary using a syringe pump. The flow rate was 5 μL min ^−1^.

### 2.5. Preparation of Detection Antibody–Fluorescent Microsphere

To prepare the detection antibody–fluorescent microspheres, a small amount of green fluorescent microspheres were added to 200 μL MES buffer solution (50 mM), and sonicated for 3 min. Then, a 6.5 μL EDC solution (5 mg mL^−1^) and a 28 μL NHS solution (5 mg mL^−1^) were added to the mixed solution and placed on a shaker bed for 60 min. After centrifuging for 20 min, green fluorescent microspheres were thoroughly cleaned with ultrapure water three times. Next, 0.2 mg of capture antibody and 2 mg of green fluorescent microsphere were added to a 200 μL HEPES buffer solution for 240 min. A 1% BSA solution was added to the mixed solution for 120 min. After the mixed solution was centrifuged, the detection antibody–fluorescent microsphere was thoroughly cleaned by HEPES buffer. Finally, the detection antibody-fluorescent microspheres were placed in a storage buffer (25 mM HEPES buffer, containing 0.1% Tween and 1% BSA).

### 2.6. Fluorescence Quantification of N-Protein

After washing the GA-modified glass capillary with PBS, 20 μL of 4 μg mL^−1^ capture antibody was injected into the GA-modified glass capillary and incubated for 20 min. Then, the remaining binding sites of the glass capillary were blocked with 10% BSA solution for 20 min. Next, the 1.37 mg mL^−1^ N-protein solution was diluted to a concentration range of 0–400 ng mL^−1^ with PBS. An amount of 20 uL of N-protein diluent was injected into a glass capillary for 15 min. After washing with PBS, 20 uL of detection antibody–fluorescent microspheres was injected into a glass capillary and combined with N-protein for 15 min. Finally, the glass capillary was placed in Smartphone-BFDD to capture fluorescence images of the N-protein diluent and to analyze its fluorescence intensity.

## 3. Results

### 3.1. Design of Smartphone-BFDD

The Smartphone-BFDD was designed using a modular assembly approach, consisting of four main modules: fluorescence detection, excitation, camera, and power. As shown in Figure 1a, the device is compact and portable, similar in size to a smartphone. The optical structure of the fluorescence detection module adopts an orthogonal design instead of a confocal design, which can reduce complex optical components and effectively shrink the size of the device. As shown in Figure 1b, the fluorescence detection module has a height of 32 mm, a width of 29 mm, and a length of 46 mm, and it consists of macrolens, camera, filter, detection chamber, optical path channel, etc., for miniaturization and integration. Orthogonal fluorescence devices, in which the excitation light has less influence on the emission light, are usually adapted for the detection of fluorescent substances where sensitivity is not required. However, biomarker detection requires fluorescence devices with high sensitivity and accuracy. To improve the detection sensitivity of Smartphone-BFDD, the design of the device is considered from two aspects: on the one hand, by minimizing the influence of reflected light and environmental light on fluorescence, and on the other hand, by maximizing the collected fluorescence from the substance being detected.

The camera module comprises a smartphone (iPhone 11) and a smartphone case, which is utilized to take pictures of a glass capillary with microchip properties. However, the proximity between the smartphone camera and the fluorescence detection module poses a problem of blurry fluorescent images due to improper focusing by the smartphone camera. In addition, the uncertainty of the location of the images captured by the smartphone also affects the subsequent fluorescence image analysis. To resolve the blurring of the fluorescent image, we added a macrolens with a magnification of 100× between the smartphone camera and the glass capillary. To test the optimal parameters of Smartphone-BFDD, BSA-FITC was selected as the test sample in subsequent experiments. As shown in Figure S2, the proper placement and distance between the three dramatically enhances the clarity of the fluorescence image. Meanwhile, we printed a smartphone case that fits both the fluorescence detection module and the smartphone, which ensures that the position of the smartphone remains constant relative to the glass capillary for easy fluorescence image analysis in subsequent steps.

The overlap of the excitation and emission wavelength ranges affects the accuracy of fluorescence image analysis. The optimum excitation and emission peaks of BSA-FITC are at 495 nm and 525 nm, respectively. To reduce the effect of the excitation light on the emission light, an LED with a wavelength of 461 nm was chosen as the excitation light source (See Figure S3). However, since LED lamps are floodlights, the light will radiate in all directions, resulting in high background light. Meanwhile, the floodlight also has weak and uneven radiation illumination intensity. To solve these problems, the Smartphone-BFDD introduces a collimating lens to convert the floodlight into parallel light. As shown in Figure S4, we ensured that the generated light spot irradiated the sample uniformly by adjusting the distance between the LED lamp, collimating lens, and the inspection chamber. Meanwhile, we observed that the light spot in touch with the glass capillary causes a strong refracted light. To reduce the interference of reflected light, we added a 470–500 nm filter to filter stray light. Furthermore, the optical path and the glass capillary were shifted from 90° to 120°, which effectively reduced the generation of refracted light (See Figure S5).

Due to the ability of black material to absorb stray light, we printed the fluorescence detection module and the power module with a black PLA material, which also reduces the interference of environmental light and stray light to some extent. The power module comprises two 1.5 V dry batteries and a battery case, which are connected in series to provide a voltage of 3 V. This dry battery is easy to replace and does not require additional charging equipment, while reducing the size, making the Smartphone-BFDD more portable and suitable for various environments.

### 3.2. Investigation of Camera Parameters for Smartphone-BFDD

RGB image analysis is a method of separating color images into three independent color channel images and analyzing their gray values, including blue image, green image, and red image. Fluorescence is light in a specific wavelength range, and it presents a stable color; quantitative fluorescent substances are realized by analyzing the gray values of the three channels. As shown in Figure S3, BSA-FITC has characteristic peaks at wavelengths between 500 nm and 550 nm. Correspondingly, BSA-FITC shows a strong green fluorescence and presents a higher gray value in the green channel image, while the excitation light is 460 nm, which presents a higher gray value in the blue channel (See Figure 2a). In Figure 2b, we can observe that the fluorescence images captured by the Smartphone-BFDD only show higher gray values in the green channel images but not in the blue channel, which proves that the design of Smartphone-BFDD is capable of minimizing the effect of the blue excitation light on the green fluorescence image. Thus, the quantitative detection of different fluorescent substances can be achieved by adjusting the parameters of excitation wavelength and filter. An example is TRITC, which has a maximum emission wavelength of 620 nm, and the analysis of fluorescence intensity can be achieved by detecting the gray value of the red channel.

**Figure 3:**
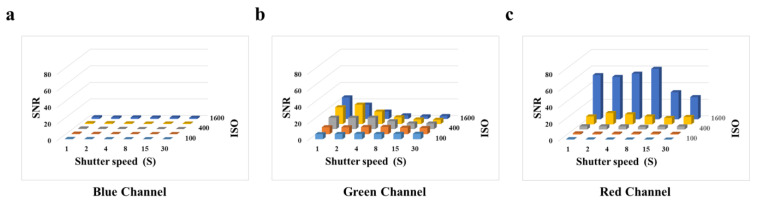
Effects of smartphone camera parameters on SNR: (**a**) Blue channel. (**b**) Green channel. (**c**) Red channel.

To evaluate the optimal parameters of the smartphone for capturing fluorescent images, we tested the effect of shutter speed and photosensitivity (ISO) on the signal-to-noise ratio (SNR) by employing the BSA-FITC as a model. According to Figure 3a, we observe that in the blue channel image, when the ISO is constant, the SNR does not change as the shutter speed increases. According to Figure 3c, we observe that the high SNR is obtained at an ISO of 1600. The gray value of the red channel image represents the light with wavelengths between 575 nm and 700 nm, which affects the gray value of the green channel image. Therefore, it is necessary to avoid the impact on other channels caused image by high ISO. According to Figure 3b, we observed that the high SNR was obtained at an ISO of 800 and a shutter speed of 2 s. However, the concentrations of biomarkers are usually very low, resulting in weak fluorescence. The shutter speed is the duration of time required for the camera shutter to open and close, which is the duration for light to enter the photosensitive component. To obtain higher fluorescence intensity in fluorescence images, it is often necessary to extend the shutter speed, which is accompanied by an increase in noise and a decrease in the SNR. According to Figure S6, we observed a significant increase in the fluorescence intensity of BSA-FITC with an increase in shutter speed, and the increase in noise was not apparent. Thus, a shutter speed from 2 s to 4 s did not significantly affect the fluorescence intensity of the fluorescence images. In consideration of this, we selected an ISO of 800 and a shutter speed of 4 s as the optimal parameters for subsequent experiments.

### 3.3. Detection of BSA-FITC Solution for Smartphone-BFDD

**Figure 4:**
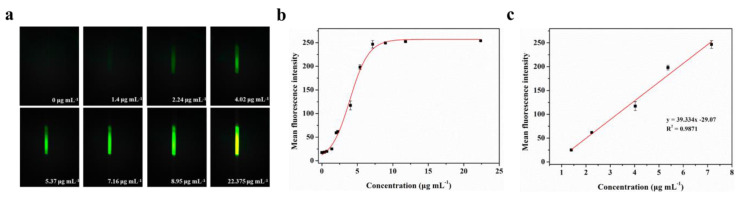
Fluorescence quantitative test of BSA-FITC: (**a**) Fluorescence images of BSA-FITC. (**b**) Fitting relationship between the mean fluorescence intensity and concentration of BSA-FITC in the range of 0–22.375 μg mL^−1^. The red line is the fitting relationship between BSA-FITC concentration and mean fluorescence intensity. (**c**) Fitting linear relationship between the mean fluorescence intensity and concentration of BSA-FITC in the range of 1.4–7.16 μg mL^−1^.

BSA-FITC is a fluorescence protein in which bovine serum albumin is directly labeled with fluorescein (fluorescein isothiocyanate, FITC) through covalent binding. To evaluate the actual detection performance of Smartphone-BFDD for fluorescent substances, we conducted a fluorescence quantitative test using BSA-FITC as a model under the optimal parameters. As shown in Figure 4a, the blank control image of the PBS solution does not show green fluorescence, which shows a low background level. As the concentration of BSA-FITC increased, the fluorescence intensity increased rapidly with the naked eye. To reduce the effect of noise generated by background superimposition on the analysis of weak fluorescence, we selected a fixed area on the captured fluorescence image and read the average fluorescence intensity within the area by utilizing image J.

A well-fitting curve between the concentration of BSA-FITC is shown in Figure 4b. We observe that the fitted curve of the mean fluorescence intensity of BSA-FITC at different concentrations shows an “S-shaped” pattern, which approaches the peak of the measurement at 11.785 μg mL^−1^. However, as we continued to increase the concentration of BSA-FITC, the mean fluorescence intensity remained unchanged. This phenomenon can be attributed to the maximum grayscale value limit of 255 in the image. Once the grayscale value of the fluorescence image reaches this limit, further increments in the concentration of the fluorescent substance no longer enable precise identification of its fluorescence intensity. The fitting linear relationship between the mean fluorescence intensity and concentration of BSA-FITC in the range of 1.4–7.16 μg mL^−1^ was obtained. After calculation, the linear equation is y = 39.334x− 29.07, R^2^ = 0.9871. The limit of detection (LOD) refers to the minimum concentration or minimum amount of the analyte that can be detected in a sample analysis. Based on the linear relationship, we can observe that the minimum detection point for BSA-FITC is 1.4 μg mL^−1^. Therefore, Smartphone-BFDD can be utilized to determine the concentration of fluorescent substances directly with the naked eye, which has the advantages of simplicity and speed. Meanwhile, the use of glass capillaries as containers for testing samples not only reduces costs but also allows the capillary phenomenon to be utilized to inject samples directly without the assistance of other instruments.

### 3.4. Characterization of the Biological Function of Glass Capillary

To achieve immobilization of capture antibodies to capture biomarkers, it is necessary to modify the inner wall of the glass capillary with functional groups that covalently bind to capture antibodies. The steps are shown in Figure S1, and the main processes are divided into hydroxyl modifications, amino modifications, and aldehyde modifications. The hydroxyl modification of the glass capillary was carried out by a two-step soaking process, which can generate a large amount of highly hydrophilic Si–OH on the surface. Compared with the equipment required for traditional plasma and ultraviolet technologies, the two-step soaking method simplifies and accelerates the entire modification process and enables large-scale one-time preparation. Then, a Si–O–Si bond is formed between Si–OH and APTES to realize the modification of the amino group. However, it is difficult for the amino group to undergo an amidation reaction with the carboxyl group of the capture antibody at room temperature. To solve this problem, we introduced GA as a linker to covalently attach the captured antibodies onto the APTES-modified layer. According to Figure S7, we observe a new peak in the 1650–1750 range after GA modification, which is attributed to the stretching and vibration of -C=O-, which confirms the success of GA modification. To verify the feasibility of the immobilization strategy, we used an electronic scanning microscope to observe the inner wall of capillaries after BSA immobilization. According to Figure S8, we used Smartphone-BFDD to observe the glass capillary immobilized with BSA-FITC and found that the surface of the capillary showed strong fluorescence, which also proved the feasibility of our immobilization strategy.

### 3.5. Detection of N-Protein for Smartphone-BFDD

**Figure 5:**
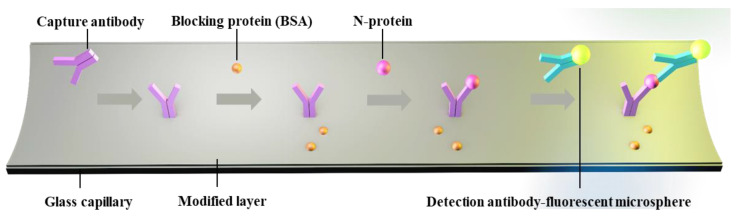
Schematic of glass capillary fluorescence immunoassay.

**Figure 6:**
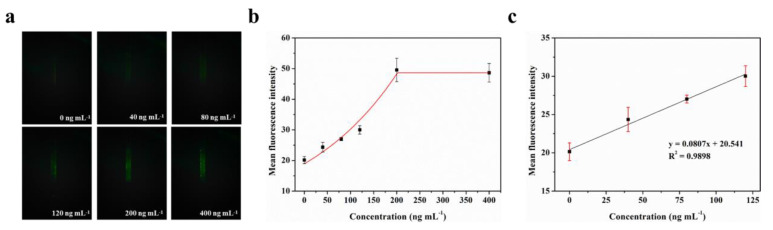
Fluorescence quantitative test of N-protein: (**a**) Fluorescence images of N-protein. (**b**) Fitting relationship between the mean fluorescence intensity and concentration of N-protein in the range of 0–400 ng mL^−1^. (**c**) Fitting linear relationship between the mean fluorescence intensity and concentration of N-protein in the range of 0–120 ng mL^−1^.

The LOD for biomarkers is crucial for early disease diagnosis. To evaluate the actual detection performance of Smartphone-BFDD for biomarkers, we used the N-protein of SARS-CoV-2 as a detection model. The level of biomarkers in the body is low, resulting in low fluorescence intensity. To improve the sensitivity of detecting biomarkers, we used a fluorescent microsphere for the detection of N-protein. Fluorescent microspheres can carry many fluorescent molecules, and a weaker stimulus can trigger a stronger signal. Following the scheme shown in Figure 5, we pre-immobilized the capture protein on the inner wall of the glass capillary, employed BSA to block any unbound site, and sequentially injected 0–400 ng mL^−1^ N-protein diluent and detection antibody–fluorescent microspheres. Ultimately, an immunofluorescent double-sandwich structure is formed on the inner surface of the glass capillary. We analyzed the fluorescence intensity of different concentrations of N-protein using a smartphone-based fluorescence detection platform and plotted the mean fluorescence curve. According to Figure 6a, as the concentration of N-protein increased, the fluorescence intensity increased rapidly with the naked eye. Similarly, we selected a fixed area of the fluorescent photo and analyzed the average fluorescence intensity of the region by utilizing image J. According to Figure 6b, the fitted curve of the mean fluorescence intensity approaches the peak of the measurement at 200 ng mL^−1^. The fitting linear relationship between the mean fluorescence intensity and concentration of N-protein in the range of 0–120 ng mL^−1^ was obtained. After calculation, the linear equation is y = 0.0807x + 20.541, R^2^ = 0.9898. Based on the linear relationship, we can observe that the minimum detection point for N-protein is 40 ng mL^−1^. The Smartphone-BFDD realized a low limit of detection (LOD, 40 ng/mL), which demonstrated the high sensitivity that can be provided for biomarker detection.

## 4. Conclusions

In summary, we developed a low-cost smartphone-based fluorescence detection device without large equipment for rapid identification and quantification of biomarkers based on glass capillary. The Smartphone-BFDD was designed using a modular assembly approach, realizing the integration and miniaturization of multifunctional modules, portable and suitable for various environments. By optimizing the Smartphone-BFDD structure and smartphone parameters, we greatly reduce the interference of excitation light on fluorescence and enhance the visualization of fluorescent images. Some scholars have constructed important smartphone-based fluorescence detection devices [37,38]. The sensitivity of fluorescence detection has been enhanced by using methods such as image stacking image analysis and improved fluorescence detection through capillary array, specifically targeting smartphones and webcams [39,40,41]. Portable detection devices involve multiple functional components of different specifications. Our system employs 3D-printing technology to print component holders, enabling the flexible and integrated assembly of multifunctional components. This approach effectively reduces the size and cost of the device, while facilitating the development of a handheld smartphone-based fluorescence detection device with broad applicability. To evaluate the performance of Smartphone-BFDD on biomarkers, we constructed an immunofluorescent sandwich structure for testing using the N-protein of SARS-CoV-2 as a biological model. Traditional SARS-CoV-2 fluorescence immunochromatographic test strips achieved fast and sensitive detection, which successfully obtained a low LOD of 100 ng mL^−1^ using 60 μL of sample [42]. Compared with these test strips, our device also has the same excellent speed and sensitivity, successfully obtaining a low LOD of 40 ng mL^−1^. This demonstrates that using this method can quickly detect the fluorescence intensity of biomarkers with only a small amount of samples (20 μL).

This work employs computer software (image J) as a means to analyze fluorescent images. However, the reliance on software for the analysis of fluorescent images (image J) is inconvenient in real-time detection. The development of smartphone software that enables integrated capture and quantitative analysis of fluorescence images will provide portability to the detection devices, allowing users to perform quick detection. In the future, we will develop RGB analysis software compatible with Smartphone-BFDD and smartphones to achieve real-time visualization and quantitative analysis. This device will inspire the detection of biomarkers and the development of novel instant diagnostic devices.

## Data Availability

All data are available in the manuscript and Supplementary Materials.

## Supplementary Materials

The following supporting information can be downloaded at: https://www.mdpi.com/article/10.3390/bios13070753/s1. Figure S1: Schematic illustrating the modification procedure of glass capillary; Figure S2: Fluorescence images of BSA-FITC with no macrolens and a macrolens; Figure S3: The excitation wavelength of LED and the emission wavelength of BSA-FITC; Figure S4: Fluorescence images of BSA-FITC with no collimating lens and a collimating lens; Figure S5: Fluorescence images of BSA-FITC at 90° and 120°; Figure S6: Fluorescent images of BSA-FITC with shutter speeds of 2s and 4s; Figure S7: Fourier transform infrared spectroscopy of glass capillary and GA-glass capitally; Figure S8: Fluorescence images of BSA-FITC with PBS solution and a BSA-FITC immobilization.

## References

[1] Crosby D., Bhatia S., Brindle K.M., Coussens L.M., Dive C., Emberton M., Esener S., Fitzgerald R.C., Gambhir S.S., Kuhn P. (2022). Early Detection of Cancer. Science.

[2] Cheung A.H.K., Chow C., To K.F. (2018). Latest Development of Liquid Biopsy. J. Thorac. Dis..

[3] Lone S.N., Nisar S., Masoodi T., Singh M., Rizwan A., Hashem S., El-Rifai W., Bedognetti D., Batra S.K., Haris M. (2022). Liquid Biopsy: A Step Closer to Transform Diagnosis, Prognosis and Future of Cancer Treatments. Mol. Cancer.

[4] Alix-Panabières C., Pantel K. (2017). Clinical Prospects of Liquid Biopsies. Nat. Biomed. Eng..

[5] Li T., Long L. (2020). Imaging Examination and Quantitative Detection and Analysis of Gastrointestinal Diseases Based on Data Mining Technology. J. Med. Syst..

[6] Niemz A., Ferguson T.M., Boyle D.S. (2011). Point-of-Care Nucleic Acid Testing for Infectious Diseases. Trends Biotechnol..

[7] Li D., Lai W., Fan D., Fang Q. (2021). Protein Biomarkers in Breast Cancer-Derived Extracellular Vesicles for Use in Liquid Biopsies. Am. J. Physiol.-Cell Physiol..

[8] Kaur M., Tiwari S., Jain R. (2020). Protein Based Biomarkers for Non-Invasive Covid-19 Detection. Sens. Bio-Sens. Res..

[9] Rifai N., Gillette M.A., Carr S.A. (2006). Protein Biomarker Discovery and Validation: The Long and Uncertain Path to Clinical Utility. Nat. Biotechnol..

[10] Cohen L., Cui N., Cai Y., Garden P.M., Li X., Weitz D.A., Walt D.R. (2020). Single Molecule Protein Detection with Attomolar Sensitivity Using Droplet Digital Enzyme-Linked Immunosorbent Assay. ACS Nano.

[11] Zhang R., Wu J., Ao H., Fu J., Qiao B., Wu Q., Ju H. (2021). A Rolling Circle-Amplified G-Quadruplex/Hemin DNAzyme for Chemiluminescence Immunoassay of the SARS-CoV-2 Protein. Anal. Chem..

[12] Claus D.R., Osmand A.P., Gewurz H. (1976). Radioimmunoassay of Human C-Reactive Protein and Levels in Normal Sera. J. Lab. Clin. Med..

[13] Mahmudi-Azer S., Velazquez J.R., Lacy P., Denburg J.A., Moqbel R. (2000). Immunofluorescence Analysis of Cytokine and Granule Protein Expression during Eosinophil Maturation from Cord Blood–Derived CD34+ Progenitors. J. Allergy Clin. Immunol..

[14] Li N., Jiang Y., Lv T., Li G., Yang F. (2022). Biosensors and Bioelectronics Immunofluorescence Analysis of Breast Cancer Biomarkers Using Antibody-Conjugated Microbeads Embedded in a Microfluidic-Based Liquid Biopsy Chip. Biosens. Bioelectron..

[15] Camara P.D., Velletri K., Krupski M., Rosner M., Griffiths W.C. (1992). Evaluation of the Boehringer Mannheim ES 300 Immunoassay Analyzer and Comparison with Enzyme Immunoassay, Fluorescence Polarization Immunoassay, and Radioimmunoassay Methods. Clin. Biochem..

[16] Lichtman J.W., Conchello J.A. (2005). Fluorescence Microscopy. Nat. Methods.

[17] Yuste R. (2005). Fluorescence Microscopy Today. Nat. Methods.

[18] Ding X., Mauk M.G., Yin K., Kadimisetty K., Liu C. (2019). Interfacing Pathogen Detection with Smartphones for Point-of-Care Applications. Anal. Chem..

[19] Wang S., Wang H., Ding Y., Li W., Gao H., Ding Z., Lin P., Gu J., Ye M., Yan T. (2022). Filter Paper- and Smartphone-Based Point-of-Care Tests for Rapid and Reliable Detection of Artificial Food Colorants. Microchem. J..

[20] Zangheri M., Cevenini L., Anfossi L., Baggiani C., Simoni P., Di F., Roda A. (2015). Biosensors and Bioelectronics A Simple and Compact Smartphone Accessory for Quantitative Chemiluminescence-Based Lateral Fl Ow Immunoassay for Salivary Cortisol Detection. Biosens. Bioelectron..

[21] Song J., Pandian V., Mauk M.G., Bau H.H., Cherry S., Tisi L.C., Liu C. (2018). Smartphone-Based Mobile Detection Platform for Molecular Diagnostics and Spatiotemporal Disease Mapping. Anal. Chem..

[22] Hussain S., Chen X., Wang C., Hao Y., Tian X., He Y., Li J., Shahid M., Iyer P.K., Gao R. (2022). Aggregation and Binding-Directed FRET Modulation of Conjugated Polymer Materials for Selective and Point-of-Care Monitoring of Serum Albumins. Anal. Chem..

[23] Nguyen H.Q., Nguyen V.D., Van Nguyen H., Seo T.S. (2020). Quantification of Colorimetric Isothermal Amplification on the Smartphone and Its Open-Source App for Point-of-Care Pathogen Detection. Sci. Rep..

[24] Chan J., Michaelsen K., Estergreen J.K., Sabath D.E., Gollakota S. (2022). Micro-Mechanical Blood Clot Testing Using Smartphones. Nat. Commun..

[25] Ghosh S., Aggarwal K., Vinitha T.U., Nguyen T., Han J., Ahn C.H. (2020). A New Microchannel Capillary Flow Assay (MCFA) Platform with Lyophilized Chemiluminescence Reagents for a Smartphone-Based POCT Detecting Malaria. Microsyst. Nanoeng..

[26] Zhao L., Wang W., Wang Y., Li H., Zhao L., Wang N., Wang Y., Wang X., Pu Q. (2021). Low-Cost Devices with Fluorescence Spots Brightness and Size Dual-Mode Readout for the Rapid Detection of Cr ( VI ) Based on Smartphones. J. Hazard. Mater..

[27] Cantrell K., Erenas M.M., Capita L.F. (2010). Use of the Hue Parameter of the Hue, Saturation, Value Color Space As a Quantitative Analytical Parameter for Bitonal Optical Sensors. Anal. Chem..

[28] Berlanda S.F., Breitfeld M., Dietsche C.L., Dittrich P.S. (2021). Recent Advances in Micro Fl Uidic Technology for Bioanalysis and Diagnostics. Anal. Chem..

[29] Faustino V., Catarino S.O., Lima R., Minas G. (2016). Biomedical Microfluidic Devices by Using Low-Cost Fabrication Techniques: A Review. J. Biomech..

[30] Morioka K., Sato H., Kuboyama M., Yanagida A., Shoji A. (2021). Talanta Quantification of CRP in Human Serum Using a Handheld Fluorescence Detection System for Capillary-Based ELISA. Talanta.

[31] Zhang T., Ding F., Yang Y., Zhao G., Zhang C., Wang R. (2022). Research Progress and Future Trends of Microfluidic Paper-Based Analytical Devices in In-Vitro Diagnosis. Biosensors.

[32] Asci Erkocyigit B., Ozufuklar O., Yardim A., Guler Celik E., Timur S. (2023). Biomarker Detection in Early Diagnosis of Cancer: Recent Achievements in Point-of-Care Devices Based on Paper Microfluidics. Biosensors.

[33] Dai B., Yin C., Wu J., Li W., Zheng L., Lin F., Han X., Fu Y., Zhang D., Zhuang S. (2021). A Flux-Adaptable Pump-Free Microfluidics-Based Self-Contained Platform for Multiplex Cancer Biomarker Detection. Lab Chip.

[34] Hussain S., Zhao H., Zhou L., Zhou X., Iyer P.K., Lv F., Liu L., Wang S. (2019). An Optoelectronic Device for Rapid Monitoring of Creatine Kinase Using Cationic Conjugated Polyelectrolyte. Adv. Mater. Technol..

[35] Wu H., Ma Z., Wei C., Jiang M., Hong X., Li Y., Chen D., Huang X. (2020). Three-Dimensional Microporous Hollow Fiber Membrane Microfluidic Device Integrated with Selective Separation and Capillary Self-Driven for Point-of-Care Testing. Anal. Chem..

[36] Gonzalez G., Roppolo I., Pirri C.F., Chiappone A. (2022). Current and Emerging Trends in Polymeric 3D Printed Microfluidic Devices. Addit. Manuf..

[37] Rasooly R., Bruck H., Balsam J., Prickril B., Ossandon M., Rasooly A. (2016). Improving the Sensitivity and Functionality of Mobile Webcam-Based Fluorescence Detectors for Point-of-Care Diagnostics in Global Health. Diagnostics.

[38] Balsam J., Bruck H.A., Rasooly A. (2013). Orthographic Projection Capillary Array Fluorescent Sensor for MHealth. Methods.

[39] Balsam J., Bruck H.A., Rasooly A. (2013). Capillary Array Waveguide Amplified Fluorescence Detector for MHealth. Sens. Actuators B Chem..

[40] Rasooly R., Balsam J., Hernlem B.J., Rasooly A. (2015). Sensitive Detection of Active Shiga Toxin Using Low Cost CCD Based Optical Detector. Biosens. Bioelectron..

[41] Balsam J., Bruck H.A., Kostov Y., Rasooly A. (2012). Image Stacking Approach to Increase Sensitivity of Fluorescence Detection Using a Low Cost Complementary Metal-Oxide-Semiconductor (CMOS) Webcam. Sens. Actuators B Chem..

[42] Zhang C., Zhou L., Du K., Zhang Y., Wang J., Chen L., Lyu Y., Li J., Liu H., Huo J. (2020). Foundation and Clinical Evaluation of a New Method for Detecting SARS-CoV-2 Antigen by Fluorescent Microsphere Immunochromatography. Front. Cell. Infect. Microbiol..

