# Supplementary material for: A Low-Cost Microfluidic-Based Detection Device for Rapid Identification and Quantification of Biomarkers-Based on a Smartphone
Source: Biosensors (Basel). 2023 Jul 22;13(7):753. doi: 10.3390/bios13070753 (PMC10377552; doi:10.3390/bios13070753)
Supplement: Supplementary file 1 [file biosensors-13-00753-s001.zip › biosensors-2436044-supplementary.pdf]

biosensors

MDPI

Supplementary Materials

A Low-Cost Microfluidic-Based Detection Device for Rapid Identification and Quantification of Biomarkers-Based on a Smartphone

Chonghui Yang †, Yujing Yang †, Gaozhen Zhao, Huan Wang, Yang Dai and Xiaowen Huang *

State Key Laboratory of Biobased Material and Green Papermaking, School of Bioengineering, Qilu University of Technology, Shandong Academy of Sciences, Jinan 250353, China; yangchonghui2022@126.com (C.Y.); 10431211246@stu.qlu.edu.cn (Y.Y.); 10431210986@stu.qlu.edu.cn (G.Z.); 10431221233@stu.qlu.edu.cn (H.W.); 10431221222@stu.qlu.edu.cn (Y.D.)

* Correspondence: huangxiaowen2013@gmail.com

† These authors contributed equally to this work.

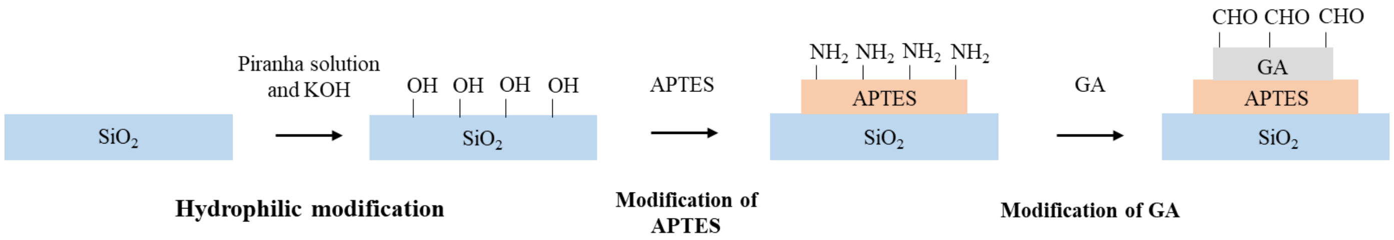

Figure S1 Schematic illustrating the modification procedure of glass capillary.

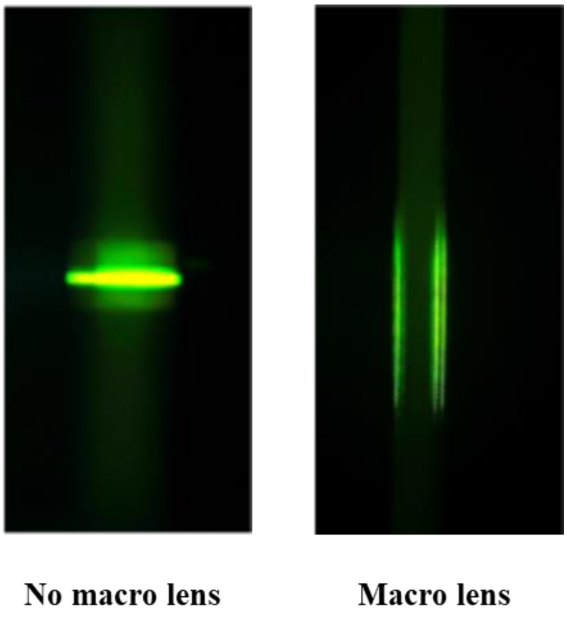

Figure S2. Fluorescence images of BSA-FITC with no macro lens and a macro lens.

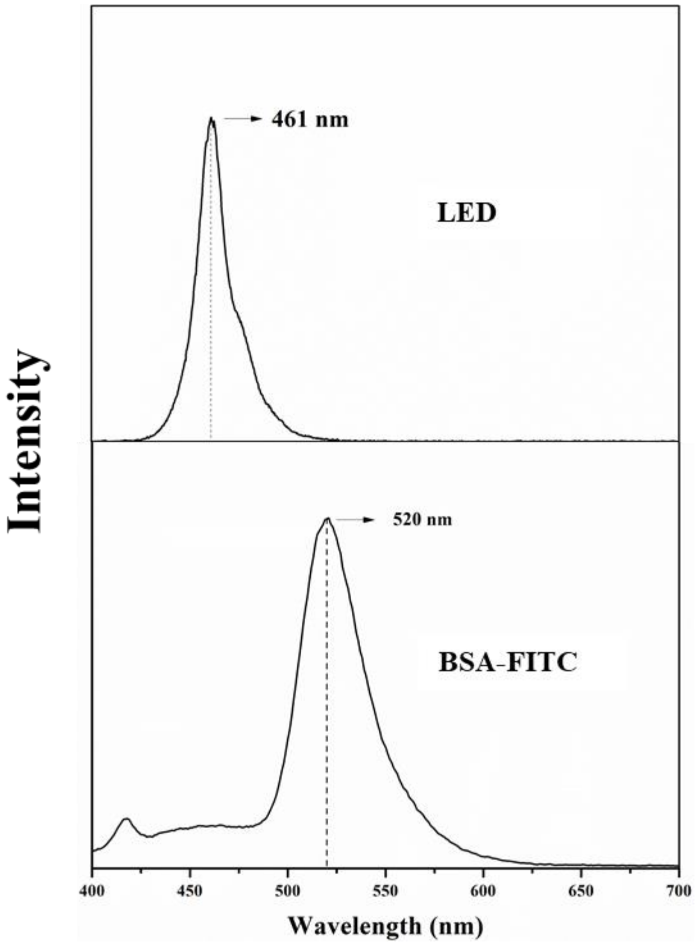

Figure S3. The excitation wavelength of LED, and the emission wavelength of BSA-FITC.

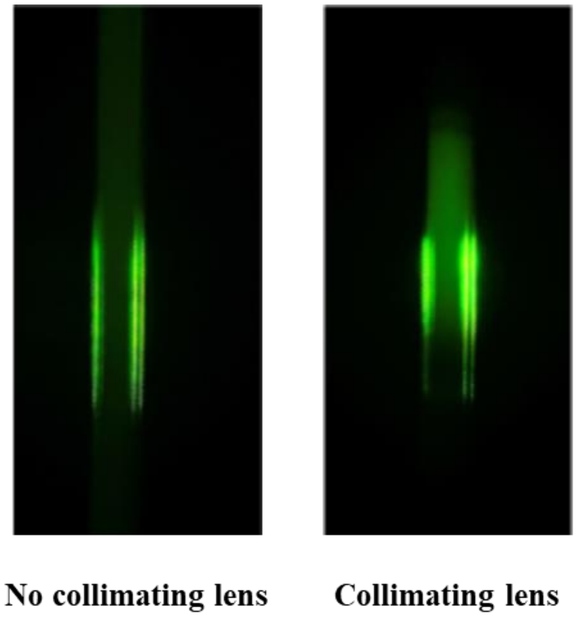

Figure S4. Fluorescence images of BSA-FITC with no collimating lens and a collimating lens.

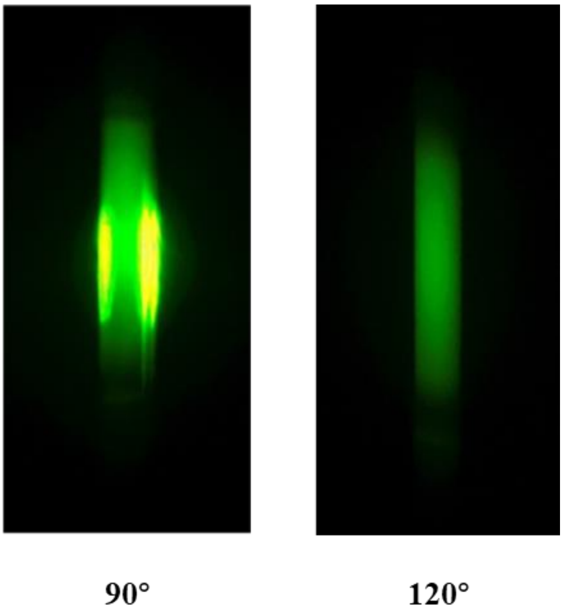

Figure S5. Fluorescence images of BSA-FITC with 90° and 120°.

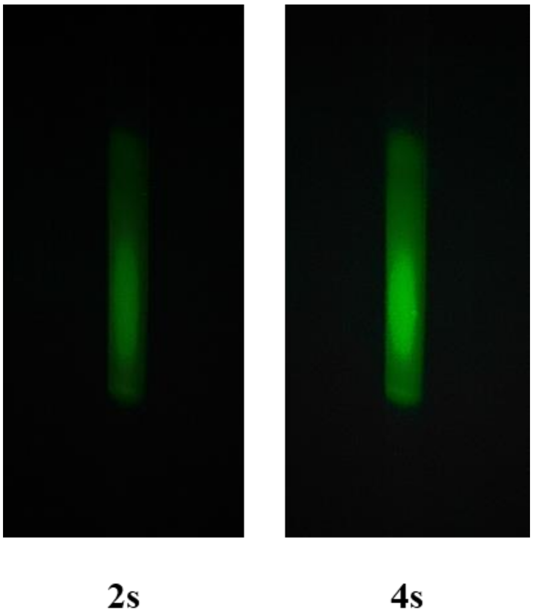

Figure S6. Fluorescent images of BSA-FITC with shutter speeds of 2s and 4s. Fluorescence intensity of BSA-FITC increases with increasing shutter speed.

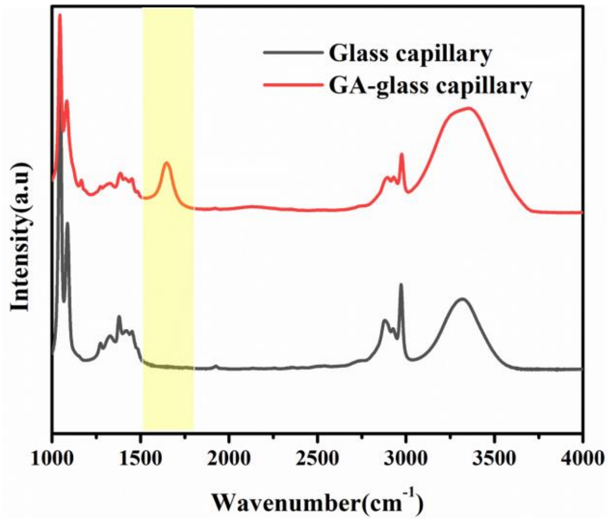

Figure S7. Fourier transform infrared spectroscopy of glass capillary and GA-glass capitally.

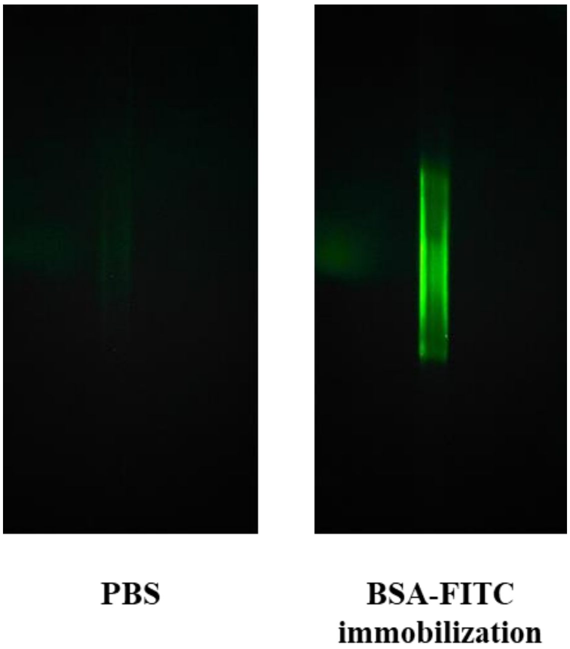

Figure S8. Fluorescence images of BSA-FITC with PBS solution and a BSA-FITC immobilization.
